# Chemotherapeutic Resistance Genes of Breast Cancer Patients - An Overview

**DOI:** 10.34172/apb.2022.048

**Published:** 2021-05-30

**Authors:** Anagha Kollamparambil Ajith, Sasikala Subramani, Agaath Hedina Manickam, Sivasamy Ramasamy

**Affiliations:** Molecular Genetics and Cancer Biology Laboratory, Department of Human Genetics and Molecular Biology, Bharathiar University, Coimbatore-641 046.

**Keywords:** Breast cancer chemotherapy, Drug resistance, Chemotherapeutic resistance genes

## Abstract

***Purpose:*** Cancer is the leading challenge to human health since the dawn of early Egyptian manuscripts, where they found tumour from fossils in the modernized 20th century. Increasing rate of incidence and death from cancer in the past few years is thought provoking. Among all type of cancers, breast cancer is very common among women and diverse in character. Drug resistance is the challenging aspect for traditional chemotherapy.

***Methods:*** Data was collected from online platform without any time restriction. After screening and evaluation, 66 articles were considered for this study. This review is a summarized collection of information from published studies on human genes associated with drug resistance in breast cancer treatment.

***Results:*** Analysis of these findings highlights the importance of MAP kinase and ABC gene families in creating resistance barriers. Genes involved in cell cycle alteration, apoptosis, and hippo pathway were also linked with drug resistance particularly in breast cancer.

***Conclusion:*** The exact mechanism of chemotherapy resistance is still unresolved and unexplained the drug resistance seen in breast cancer patients were multifactorial. Drug induced up regulation or down regulation of genes contributes unusual protein expression and ultimately leads to resistance. The ultimate focus of this review is to identify the genes having pivotal role in chemotherapy resistance in breast cancer.

## Introduction

Abnormal growth of cells due to uncontrolled cell proliferation in body is called as cancer.^1^ Breast cancer is one of the world’s commonest cancers with a collection of neoplastic diseases that are molecularly and clinically complicated and comprise different sub types with unique characters.^2^ Breast cancer is rarely seen in males.^3^ Being a highly prevalent cancer type, it is observed in both developing and developed countries with different causes and factors of progression. Adopting new life styles, industrialization, globalization, increased life expectancy, pollution etc are the leading cause of cancer. 2012 Global Cancer statistics reveals that among the eight million diagnosed cases of cancer all over the world, one million constitute of breast cancer.^4^ According to 2015 cancer statistics almost 231 000 US women were diagnosed and about 40 000 patients were died with breast cancer. As per 2018 statistics, every year around 1.2 million new cases of breast cancer were diagnosed.^5^ The GLOBACON report of 2018 concluded breast cancer as the most frequently identified cancer type and leading cause of cancer related death in majority of the countries.^6^ Major part of breast related cancer are linked with the expression of estrogen receptor and their treatment is related with the disease prognosis.^5^

Rate of breast cancer mortality can be reduced up to a limit by early diagnosis; timely treatment and management.^6^ commonly adopted treatment strategies for breast cancer are chemotherapy, radiation therapy and hormone therapy. Among them chemotherapy is widely accepted as traditional treatment method, in spite of its obstacle due to chemotherapy resistance. Important mechanisms lead to drug resistance includes alteration in expression of ABC transporters gene family, damage of topoisomerase enzyme, mutation in DNA repair genes, induced apoptosis by genetic imbalance, alteration in signalling pathways of NF alpha etc. All these worsen the condition of breast cancer making chemotherapy a failure in most cases.^7^ The cells which already acquired drug resistance show cross resistance to anti proliferative nature of anti-oestrogen drugs which have crucial role in breast cancer treatment.^1^ In this review, we are discussing on various genes involved in chemotherapy resistance pathways of breast cancer.

###  Genes involved in MAP kinase pathway

Mitogen activated protein kinase (MAPK) is a group of protein which communicates and transfers signal from the cell surface to the nuclear environment in response to external stimuli.^8^ Alteration in MAPK pathway cause poor tamoxifen response in ductal carcinoma and estrogen expression in lobular breast cancer. Copy number alteration and mutation acquired by drug resistant cells affect the metallotropic glutamate receptor and MAPK pathway. MAP deregulation induces tamoxifen resistance. Likewise the hyperactivity of MAPK is closely linked with *GRM1* gene mutation.^9^ The *GRM1* gene is considered as an oncogene in epithelial cells.^10^ Un controlled expression of the *KLF4* gene, plays an important role in the transition of G1 to S phase through P53. Phosphorylation of MAPK pathway and estrogen receptor kinase and *P38* activation were induced by *KLF4* knock down causing tamoxifen resistance. Hyperactivity of MAPK signalling by the *c- ras, b- raf, MEK* ½mutation is commonly seen in many human cancer cells including breast cancer.^11^ MAPK/ERK pathway regulates cell cycle and cell proliferation having a crucial role in cancer treatment especially in triple negative breast cancer cases.^12^ Heterodimerization of RAF kinase cause the activation of ERK pathway and drug resistance. Demodulation of *Jak*, *stat* 3 and *Akt* signalling along with MAP kinase pathway cause paclitaxel resistance by *STAT3* in breast cancer,^13^ and also the basal activity of JAK1/STAT1 and JAK1/STAT3 signalling is higher in chemo resistant cells. The Genes of STAT3 Inhibition involved in apoptosis activation is down regulated in chemotherapy resistant cells.^14^

###  Genes associated with drug efflux

Efflux proteins are involved in the transportation of drugs and toxic substance from inside the cell to the outside ATP dependent drug efflux pumps may sometimes reduce drug uptake. *ABCD1*, *C1*, and *G2* transporters are extensively studied ABC transporters gene family involved with drug resistance.^15^

####  The ATP-binding cassette (ABC) transporters

*ABCG2* gene is commonly known as *BCRP* which is a part of ATP binding cassette transporters that play a major role in cellular transportation by the expense of ATP across concentration gradient inducing drug resistance mechanism.^16,17^ various anti cancers agents’ effluxed by altered expression of p-glycoproteins are considered most studied and explained mechanism of drug resistance. P-glycoprotein is an example of adenosine triphosphate binding cassette protein, which expressed by the mutation of genes in ABC family and drug doxorubicin, acquires resistance through the mutation of this gene family. This in turn because drug effects leading to insufficient accumulation of doxorubicin in nucleus, showing only about 0.4% doxorubicin enter cell after internalization.

Resent research points out multiple drug resistance induced by oxidized low density lipoproteins and very low-density lipoproteins. Down regulation of p-glycoproteins by these lipoproteins induce alteration in *ABCB1* gene expression, paving the way for multiple drug resistance through drug efflux. Many study reveals that the cells treated with epirubicin and anthracycline, show *ABCG2* activation and expression in breast cancer cell lines by self-renewing capacity. *ABCG2* gene has an important role in gefitinib resistance. Which is used for *EGFR* over expressed breast cancer like erlotinib. These drugs interrupt *EGFR* signaling in the target cells. Drug induced over expression of proteins by *ABCG2* genes acquire resistance to the drug gefitinib in EGFR expressed breast cancer cells.^18^ At low concentration gefitinib act as a substrate for ABCG2 protein, inducing gefitinib resistance through *Akt* nuclear EGFR (nEGFR) pathway.^19^ *ABCI* gene family induce drug efflux causing resistance to methotrexate,^20^ and up regulation of *GPR120* gene cause alteration in GRP120 protein mediated signaling, inducing resistance to epirubicin in chemotherapy, and Akt NFKB signaling is responsible for *GRP120* mediated resistance to epirubicin.^21^ Intracellular epirubicin accumulation is directly associated with ABC transports expression *ABCG1* and *ABCG2* gene up regulation by NF-kB p65 cause blockage of GPR120 signaling.^22^ *FASN* and *ABCG2* expression is decreased by GPR120-si-RNA and *GP6120* expression is related with the levels of AACC1, ABCC2, FASN and FFAs. All these are directly or indirectly related to ABC gene up regulation and multiple drug resistance.

####  Multiple drug resistant genes

ABC gene family members MDR1 have a significant role in drug resistance.^23^ MDR1 over expressions by p53 alteration cause chemo resistance in doxorubicin and Tuxol. Which significantly contributes changes in P-glycoprotein levels there by inducing resistance.^24^ Acquired and intrinsic cross resistance to vinca alkaloid derived drugs and anthracycline are related to p glycoproteins induced *MDR1* expression.^25^ Deletion of the *mir 125b* gene in chromosome 19q and microRNA 451 regulate *MRD1*expression in anthracycline induced resistance.^26^

###  Cell cycle alternating genes

Chemotherapy cause changes in genes involved in cell proliferation by holding the cell division, where FOX protein family have a significant role in regulating cell cycle. Studies report over expression of the androgen receptor as a reason for chemotherapy resistance, contributes uncontrolled cell proliferation in triple negative breast cancer patients.^27^ FOXA1 is an inhibitor of AR signaling by detecting it is binding to the cells, whose activity completely overlaps estrogen receptor binding site in an AR positive cells lines and cause tamoxifen resistance. GRLH2 binding site is closely associated with FOXA1 binding site.^12^ Tamoxifen resistance result in LYPD3 protein level elevation, which is considered as a target for GRHL2.^28^ One of the reasons for *HER2* mediated drug resistance is by the amplification or deletion of Topoisomerase II alpha gene *TOP2A located* in 17q21 near to ERBB2 oncogene.^29^ Topoisomerase II alpha is the molecule target for topo II inhibitors, potent anti-cancerous drug.^30^

###  Genes mediated drug resistance through preventing apoptosis

Apoptosis pathways have a significant role in cancer treatment, and any alteration in this pathway is a major obstacle for effective treatment.^31^ Two routes are involved in the activation of apoptosis cascade. The intrinsic pathway also known as mitochondrial pathway that releases cytochrome C by activation of tumor necrosis factor in response to ligand binding.^32^ Autophagy, mitotic catastrophe, necrosis, and senescence are non-apoptosis mechanism involved in cell death.

*CASP3* gene is located in the 4q34 with size 2635 bp, this mediates apoptosis in response to chemotherapy, but losing caspase 3 activity cause cell survival and induce drug resistance through apoptotic pathway in breast cancer.^33^

*K1F14* is a tumor inhibitor gene that prevents cell migration and induce cell apoptosis and its knockdown is found increases breast cancer subjects.^34^ *KIF14* plays an important role in drug resistance by enhancing cell proliferation through *Akt1* pathway which with decreased activation lowers the level of *Akt* and affects the *P13K/ Akt* signaling that controls the apoptosis pathway. It also decreases the levels of tumour suppressor gene *p63,*^35^ promoting *Akt* phosphorylation. Docetaxel is one of the frequently used drugs for breast cancer and decreased *KIF14* expression increase docetaxel resistance.^36^ *FOXM1* is a cell proliferation gene which encodes a protein regulating cell cycle gene and also an apoptosis inhibitor. *XIAP* produces protein for preventing apoptosis and survivin is produced by BIRC5, whose expression covers upregulation in drug resistance. *XIAP* and *BIRC5* induce taxane and anthracycline resistance.^37^ Survivin, induce multiple drug resistance to drugs in tumors associated with endothelial cells causing resistance to drugs like paclitaxel and temozolomide by preventing apoptosis of caspase 7 and 4.^38^

O6 methyl guanine methyl transferase is a DNA repair enzyme, whose increased activity causes resistance to drug such as temozolomide, streptozotocin, procarbazine and dacarbazine. This induces DNA lesions.^39^ The genes *XRCC2* and *BRCA2* involved in homologous recombination and DNA damage repair. This confers cisplatin induced drug resistance by maintaining DNA damage.^19^ *XRCC2* stimulates *RAD51* levels and Elevated RAD51 level is linked with high recombination rate and increases resistance to DNA altering drugs.^40^ *XRCC2* expression is directly linked with drug resistance by different mechanisms.^41^

**Figure 1:**
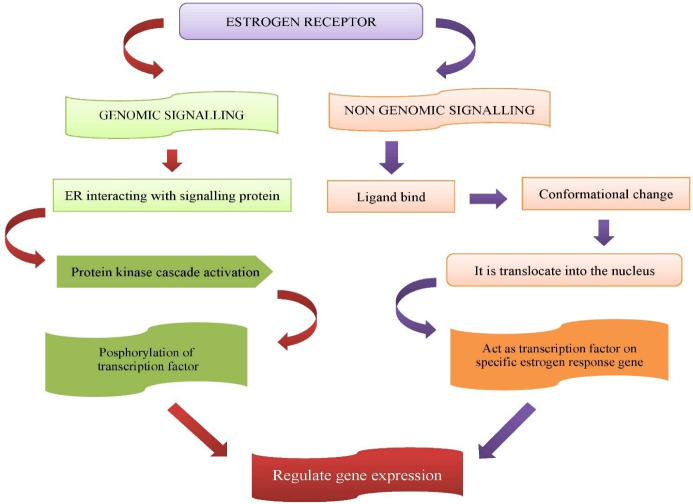
Tamoxifen regulates estrogen modulation through non genomic and genomic signalling

*ASK1* gene is a *MAP3 K* member which plays a vital role in breast cancer cell apoptosis under stress conditions. Raf 1 mediated inhibition of *ASK1* cause drug resistance in endothelial cells through basic fibroblasts growth factor. Pro apoptotic activity of this gene is inhibited by BFGF, which leads to the end of apoptosis.^42^ *ASK1* gene’s level is related with *CLDN6* expression and over expression of *CLDN6* is linked with breast cancer chemo resistance through GSTP1 its activity is regulated by *p53.*^43^ in the treatment of hormone sensitive breast cancer, tamoxifen resistance is a clinical obstacle. The major genes involved in tamoxifen resistance by X box binding proteins. Which is directly related with estrogen receptor alpha function. First three amino acids of the protein coding gene involved in the activation of estragon receptor in estrogen deficiency.^12^ Tamoxifen also targets the mitochondrial genes like *SIRT3* and *AMPK.* The protein encoded by *SIRT3*regulats ATP generation, aging and carcinogenesis. It has a significant role in inhibiting apoptosis and recent studies show the evidence of this gene in drug resistance.^44^ this drug up regulates the *SIRT3* gene function and *AMPK* gene phosphorylation. Where *AMPK* has a crucial role in drug resistance by producing self-renewal cancer stem cells.^45^ AMPK also act as a downstream regulator of tumour suppressor genes like *P53* and *LKB1*. Tamoxifen regulates estrogen modulation through non genomic and genomic signalling^46^ (Figure 1).

**Figure 2:**
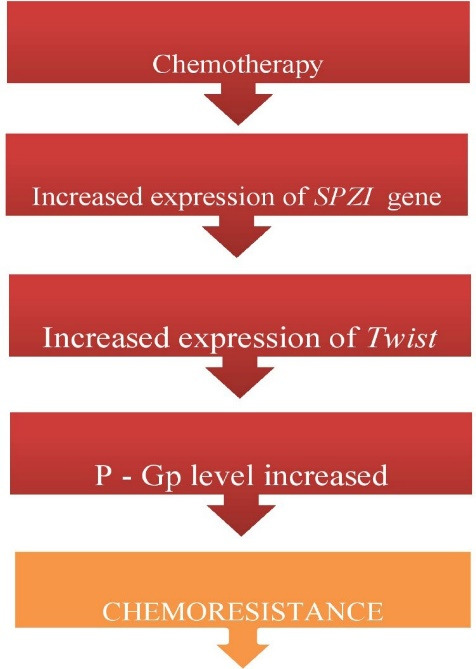
Crucial role of SPZ1 in chemotherapy resistance through TWIST gene alternation

Chemotherapy increases the expression of *BHLH* genes. Anti-cancerous drugs which acts on Microtubule in a disruptive manner induces drug resistance by up regulation of *TWIST* gene.^47^ Likewise knockdown of SPZ1 have a crucial role in chemotherapy resistance through TWIST gene alternation^48^ (Figure 2).

The anti-cancer drugs used for DNA repair can induce drug resistance through the same mechanism, whereas DNA mismatch repair gene like *MSH2* and *MLH1*causes resistance to topoisomerase II inhibitor doxorubicin and mitoxantrane.^49^

###  Drug inactivation

Drug inactivation phenomenon refer to the decreasing amount of free drug available in the cell to be detected and bind to a particular intracellular target. Chemotherapeutic Platinum drugs covalently links to glutathione related enzymes and promote drug efflux by acting as a substrate complex for the ABC transporters protein.^50^ The glutathione linking is catalyzed by the glutathione-transferase enzyme family. High level of GST- π is a reason for cisplatin induced drug resistance.^51^ Where P glycoproteins related with GST II also causes multiple drug resistance. Paclitaxel cause over expression of *GSTII*^52^ and the GST family protein is considered as a bad prognostic indicator for drug resistance.^53^

###  Cancer stem cells

**Figure 3:**
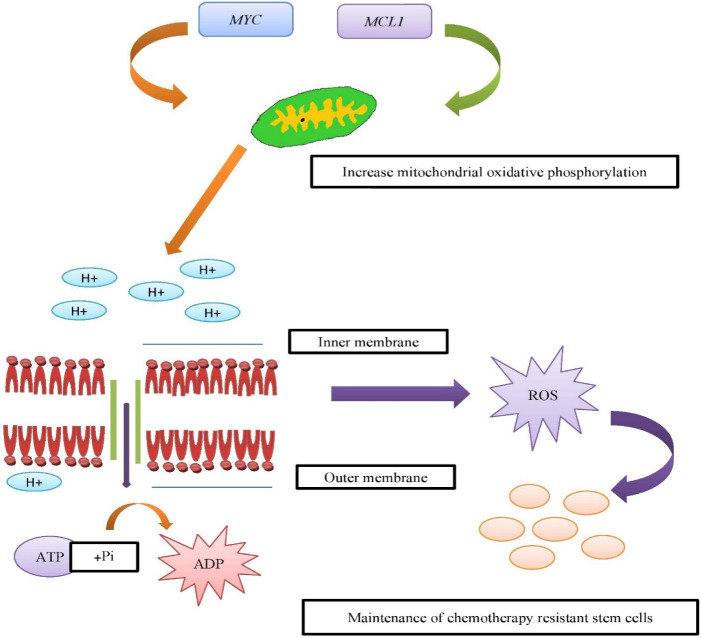
Upregulation of ROS production in Breast cancer.

The knowledge of cancer stem cell opens a new insight in the study of oncogenesis and cancer treatment.^54^ The characteristic features of the stem cells differentiate it from normal cells and in addition to its self-renewal property it can stay in a state of dormancy and infrequent division. Requiring special environmental conditions for division.^55^ *ABC* transporters gene family have a significant role in chemotherapeutic agents induced formation of cancer stem cells in breast cancer. *ABCG2* and *ABCB1* are the important genes involved in the formation of cancer stem cells^56^ and *MYC* and *MYCL* are another gene involved in drug resistance by different mechanism and most important method is promoting colony formation capacity.^57^ Stem cells which is formed as a result of chemotherapy cause colonization of cancer at different places. *MYC* carries out cell proliferation and apoptosis; in *TP53* mutation *MYC and MYCL* are co amplified.^58^ This directly results in the breast cancer stem cells enrichment by enhancing mitochondrial respiration. It also upregulates ROS production in breast cancer (Figure 3).

###  Genes associated with hippo pathway

Hippo pathway is identified first in Drosophila Melanogaster, and later identified in mammals. Core components are Kinase *MST1/2* (mammalian Ste-20 like kinase), LAST 1/2 (large tumour suppressor), *YAP* (yes associated protein), TAZ (transcriptional co activator with PD2), *RASSF1.* Hippo pathway is activated by *MSTA1/2*. Transcription of *YAP* and *TAZ* are inhabited by the phosphorylation and down regulation of *LAST1/2*. Hippo pathway activation is closely related with breast cancer stem cell formation.^59^ Breast cancer stem cell’s improper *TAZ* and *YAP* activity cause multiple drug resistance. An anti-Microtubule drug taxol cause *LAST2* knockdown. *LAST2* play vital role in up regulating estrogen receptor, and this cause resistance to tamoxifen, Studies shows that high level of *TAZ* were observed in breast cancer cells.^60^

###  Vault proteins associated genes

Vaults are largest nucleoprotein particle ever discovered which have a size of ~42 × 75 nm and ~13 MDa mass. It is first described in 1986.^61^ Studies suggest that *MVP* protein have a particular role in drug resistance through the activation of *MAP* kinase pathway. *MYP* is the primary component of vault complex. It is involved in chemotherapy resistance by phosphoinositide-3-kinase/*AKT* signalling and EGFR induced *MAPK* pathway associated with *ABCC1* and *ABCB1*. *NOTCH1* down regulate *MVP* expression.

## Discussion

**Figure 4:**
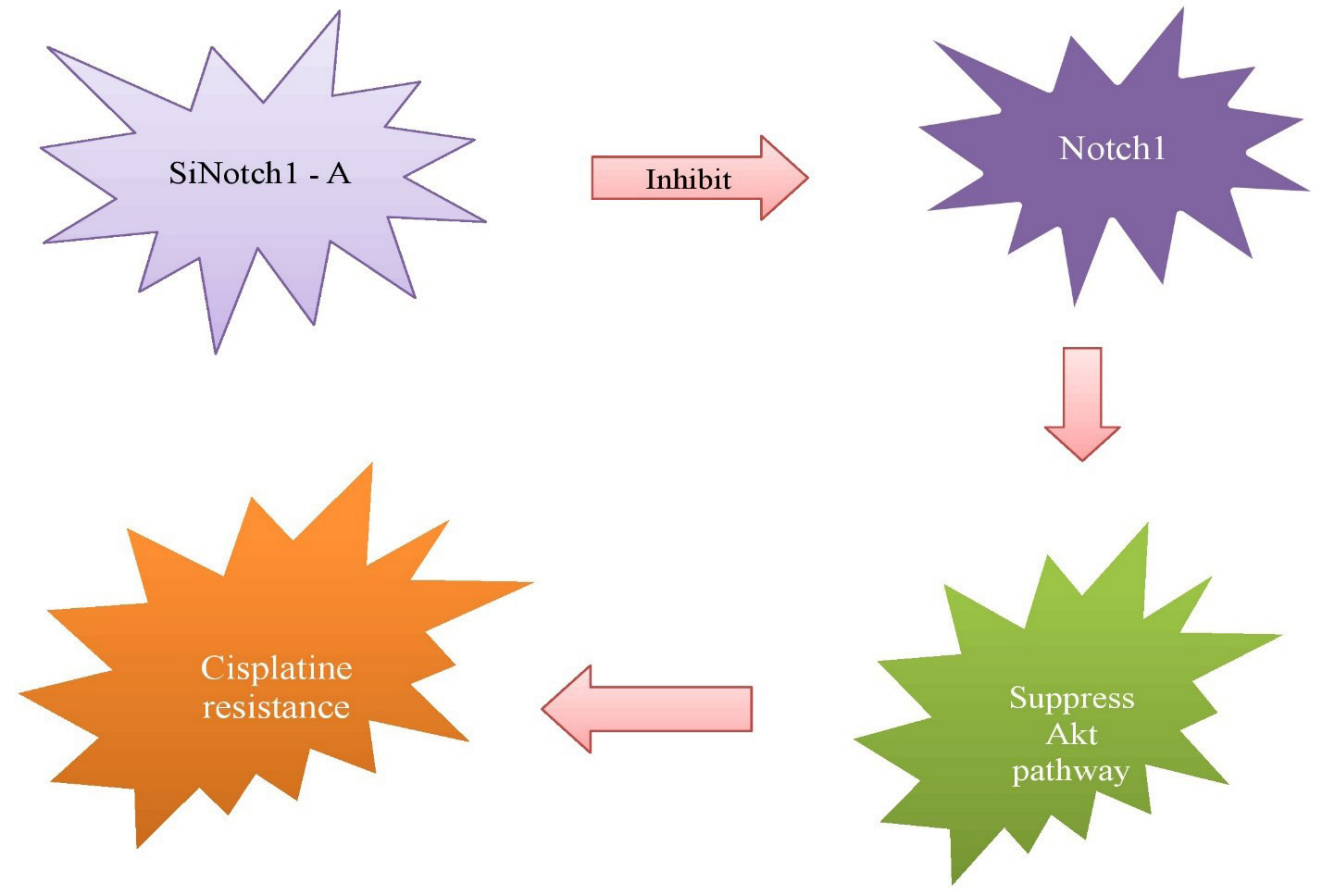
the amalgamation of anti-cancer drugs

Different genes play a crucial role in resistance to multiple drugs (Table 1). Genes related with MAP kinase pathways which include, *GRMI, KLF4* etc have clear link with drug resistance in breast cancer. *KLF4* knockdown cause tamoxifen resistance and *STAT3*cause paclitaxel resistance. Another important members involved are ATP-binding cassette (ABC) transporters gene family which include *ABCB1, ABCG2* and Multiple drug resistant gene family like *MDR1* genes. Chemotherapy resistance induced by preventing apoptosis is mediated by genes like *FOXM1, XIAP, BIRC5, XRCC2, BRCA2, CLDN6, ASK1, SIRT, AMPK, TWIST* etc. Genes associated with hippo pathway and vault proteins associated genes induce drug resistance by various mechanisms. Conventional cancer chemotherapy is severely restricted by multidrug resistant tumor cells due to changes in the level or activity of membrane transporters that mediate energy dependent drug efflux and proteins that influence drug metabolism. The extensive use of chemotherapeutic agents for cancer treatment has led to many patients being cured. Sadly, many cancers do not yet respond to chemotherapy, and other cancers which initially respond later become resistant. The use of biologics and gene therapy is a highly active area of research to address the problems of chemo resistance. Combination therapy, a type of treatment that incorporates two or more therapeutic agents, is one of the cornerstones of cancer treatment. Similar to the monotherapy strategy, the amalgamation of anti-cancer drugs increases effectiveness because it addresses main pathways in a synergistic or additive way. (Figure 4).

**Table 1:** Most studied genes involved in drug resistance in breast cancer

**Gene**	**Important drugs**	**Mechanism**	**References**
*GRM1*	Doxycycline	MAP kinase pathway alternation	^11^
*KLF4*	Tamoxifen	MAP kinase pathway alternation	^13^
*STAT3*	Paclitaxel	Alternation in apoptosis pathway	^16^
*ABCG2*	Doxorubicin, epirubicin, Anthracycline, gefitinib, erlotinib methotrexate	Drug efflux	^23,21^
*ABCB1*	Cisplatin	Drug efflux	^62^
*GPR120*	Epirubicin, anthracycline	Drug efflux	^2^
*MDR1*	Paclitaxel	Alternating androgen receptor signaling	^26^
*FOXA1*	Tamoxifen		^11^
*TOP2A*	TOPOII Inhibitors	Cell cycle alternation	^32^
*CASP3*	Epirubicin, paclitaxel	Alternating apoptosis signaling	^7^
*KIFI4*	Docetaxel	Enhance cell proliferation	^63^
*FOXM1*	Tamoxifen, anthracycline	Induce uncontrolled cell division	^38^
*XRCC2*	Cisplatin	Apoptosis pathway	^27^
*STRT3*	Taxol	Preventing apoptosis	^45^
*AMPK*	Taxol	Apoptosis pathway alternation	^47^
*GSTπ*	Cisplatin, paclitaxel	Drug inactivation	^52^

## Conclusion

 The drugs combinations which are currently exist were refined from clinical experiences and it comprises a small part of therapeutic space. Computational approach of the biological science to discover new collaborative drug combinations for treating breast cancer will help in overcome multiple drug resistance, rather than preventing drug resistance, it also prevents cell’s mitotic activity, suppress the proliferation of cancer stem cells, reduce the rate of tumor formation and induce apoptosis. The five- year survival rates for most metastatic cancers are still quite small, so developing a new anti-cancer medication is costly and extremely time- consuming. Optimization of therapeutic efficacy by taking into account the therapeutic synergy that is immune to multiple drug dosing and scheduling can be carried out to overcome the acquired drug resistance in breast cancer patients.

## Ethical Issues

 No ethical issues are associated with the publication of this review article.

## Conflict of Interest

 Not applicable.
